# Residual Foci of DNA Damage Response Proteins in Relation to Cellular Senescence and Autophagy in X-Ray Irradiated Fibroblasts

**DOI:** 10.3390/cells12081209

**Published:** 2023-04-21

**Authors:** Andrey Osipov, Anna Chigasova, Elizaveta Yashkina, Maxim Ignatov, Yuriy Fedotov, Daria Molodtsova, Natalia Vorobyeva, Andreyan N. Osipov

**Affiliations:** 1 N.N. Semenov Federal Research Center for Chemical Physics, Russian Academy of Sciences, 119991 Moscow, Russia; a-2-osipov@yandex.ru (A.O.); annagrekhova1@gmail.com (A.C.); yashkinaliz@gmail.com (E.Y.); mantroz@yandex.ru (M.I.); ufedotov456@gmail.com (Y.F.); nuv.rad@mail.ru (N.V.); 2 Emanuel Institute for Biochemical Physics, Russian Academy of Sciences, 119334 Moscow, Russia; 3 State Research Center—Burnasyan Federal Medical Biophysical Center of Federal Medical Biological Agency (SRC—FMBC), 123098 Moscow, Russia; dmolodtsova@gmail.com; 4 Joint Institute for Nuclear Research, 141980 Dubna, Russia

**Keywords:** DNA double strand breaks, DNA damage response, residual DNA repair foci, cellular senescence, autophagy, caspase-3, proliferation, fibroblasts, X-ray radiation

## Abstract

DNA repair (DNA damage) foci observed 24 h and later after irradiation are called “residual” in the literature. They are believed to be the repair sites for complex, potentially lethal DNA double strand breaks. However, the features of their post-radiation dose-dependent quantitative changes and their role in the processes of cell death and senescence are still insufficiently studied. For the first time in one work, a simultaneous study of the association of changes in the number of residual foci of key DNA damage response (DDR) proteins (γH2AX, pATM, 53BP1, p-p53), the proportion of caspase-3 positive, LC-3 II autophagic and SA-β-gal senescent cells was carried out 24–72 h after fibroblast irradiation with X-rays at doses of 1–10 Gy. It was shown that with an increase in time after irradiation from 24 h to 72 h, the number of residual foci and the proportion of caspase-3 positive cells decrease, while the proportion of senescent cells, on the contrary, increases. The highest number of autophagic cells was noted 48 h after irradiation. In general, the results obtained provide important information for understanding the dynamics of the development of a dose-dependent cellular response in populations of irradiated fibroblasts.

## 1. Introduction

The study of the dose–response relationships is imperative in radiation therapy to assess the mechanisms of organ and tissue response to ionizing radiation (IR). When patients receive radiation therapy it is necessary to evaluate the expected response level in normal tissues, since the goal is to destroy the tumor while reducing the damage to healthy normal tissues as much as possible. Usually, the dose to the tumor is determined by the tolerance of the surrounding normal tissues. It is important to know exactly which biological processes occur in tissues to infer a model that can accurately describe the normal tissue response and determine tissue tolerance in different situations. This knowledge will help to predict the quality of life after radiation therapy by estimating the probability of tumor cure and the risk of treatment related-morbidity. Modeling the response of healthy tissue to radiation has become an important domain of modern radiation therapy [1]. Radiobiological studies at the molecular and cellular levels help to uncover the damage mechanisms that operate when cells or organisms are exposed to IR. Indicators of cellular damage include identification of DNA damage, chromosome aberrations, cell survival, etc.

Among a wide range of radiation-induced DNA damage types (base modifications, abasic sites, single- and double-strand breaks, DNA–DNA and DNA–protein cross-links), double-strand breaks (DSBs) are the most critical for the further fate of an irradiated cell [2]. In response to the formation of DSBs in cells, the DNA damage response (DDR) is activated, which includes DNA repair, cell cycle arrest, programmed cell death, and loss of the cell’s ability to divide (cellular senescence). Relatively correct DSB repair by the mechanism of homologous recombination (HR) requires the presence of sister chromatid DNA as a template and, therefore, is possible only during the relatively short S and G2 phases of the cell cycle [3]. As a result, most of the DSBs in the irradiated cell are eliminated by the mechanisms of classical non-homologous end joining (NHEJ), alternative end joining (A-EJ), microhomology-mediated end joining (MMEJ) and single-strand annealing (SSA) [3,4]. As a result of incorrect DNA repair, especially by the A-EJ and MMEJ mechanisms, microstructural chromosome aberrations and various cytogenetic disturbances (stable and unstable chromosomal rearrangements, micronuclei) occur, while the impossibility of repair leads to the launch of cell death or cellular senescence mechanisms. At present, there is no doubt that incorrect DNA DSB repair, especially by the A-EJ and MMEJ mechanisms, is one of the main causes of genome instability, carcinogenesis, and aging [5,6,7].

DDR is a complex process involving dozens of proteins, differentiated by their functions into sensors, transducers, mediators and effectors. The DDR signal has the ability to be amplified. For example, such a DSB sensor as MRE11/RAD50/NBS1 (MRN) activates the transducer kinase ATM (ataxia telangiectasia mutated), inducing its autophosphorylation, ATM, in turn, rapidly phosphorylates the core histone H2AX, facilitating chromatin binding with the mediator of damage-checkpoint 1 (MDC1) [3]. MDC1 interacts with MRE11 and the cycle repeats. As a result, in the chromatin regions flanking the DSB, complex dynamic microstructures are formed, consisting of proteins involved in DDR with different copy numbers (up to several thousand copies). With immunocytochemical staining, such protein microstructures, which are called DNA damage or DNA repair foci in the literature, are easily visualized with microscopy [8,9]. The patterns of formation and elimination of radiation-induced foci of the phosphorylated histone H2AX (γH2AX) [10,11], phosphorylated ATM (pATM) [12,13] and the mediator protein—p53 binding protein 1 (53BP1) [14,15]—have been particularly well studied, whereas the works devoted to the analysis of radiation-induced foci of phosphorylated effector protein p53 (p-p53) are rare [16]. The kinetics of post-radiation changes in the amount of DNA repair foci differ significantly depending on the cell type, cell cycle phase, linear IR energy transfer, irradiation regimen and dose. In the case of human fibroblasts singly irradiated with X-rays at doses of 100–1000 mGy, the maximum number of γH2AX foci is observed 30–60 min after irradiation, followed by their decrease by ~50% within 4–6 h, and after 24 h they decrease by up to 85–95% of the amount recorded at the maximum point [17]. DNA repair foci observed 24 h and later after irradiation are called “residual” in the literature [18]. They are believed to be the repair sites for complex, potentially lethal DNA DSBs [19,20,21,22,23]. It has been shown that an increase in the number of residual foci is associated with a decrease in colony-forming ability [23]. The reason for the decrease in clonogenic growth and survival is not only cell death, but mainly the loss of the ability to divide due to cellular senescence. However, the features of their post-radiation quantitative changes and their role in the processes of cell death and senescence have not been sufficiently studied yet.

The aim of this work was to investigate the relationship between the changes in the number of residual DNA repair foci of key DDR proteins (γH2AX, pATM, 53BP1, p-p53) and the fractions of senescent, caspase-3 positive and autophagic cells in human fibroblasts 24, 48 and 72 h after exposure to X-rays at doses of 1–10 Gy. The results of this study will help us to move forward in modelling the response of healthy cells to IR.

## 2. Materials and Methods

### 2.1. Cell Culture

Human dermal fibroblasts (Cell Applications, San Diego, CA, USA, Catalog Number: 106K-05a) were cultured in DMEM culture medium with high glucose content (4.5 g/L) (Thermo Fisher Scientific, Waltham, MA, USA) supplemented with 2 mmol/L L-glutamine (Thermo Fisher Scientific, Waltham, MA, USA), 100 U/mL penicillin, 100 μg/mL streptomycin (Thermo Fisher Scientific, Waltham, MA, USA) and 10% fetal bovine serum (Thermo Fisher Scientific, Waltham, MA, USA). Cell cultivation was performed under the standard conditions of a CO_2_-incubator (37 °C, 5% CO_2_, saturated humidity). For irradiation experiments, cells of the 5–7th passages were used.

Prior to irradiation, the cells were seeded at a density of 0.4 × 10^5^ cells/mL in 2.5 mL of culture medium onto coverslips (Thermo Fisher Scientific, Waltham, MA, USA) placed inside 35 mm Petri dishes (Corning, New York, NY, USA) and incubated at 37 °C and 5% CO_2_ for 20 h. Cells were irradiated in the phase of exponential growth (cell population density ~60%).

### 2.2. Irradiation

Cells were irradiated using a RUB RUST-M1 X-ray unit (Diagnostika-M LLC, Moscow, Russia) equipped with two X-ray emitters at a dose rate of 0.85 Gy/min (voltage of 200 kV, an anode current of 2 × 5 mA, and a 1.5 mm Al filter). After irradiation, cells were incubated under the standard conditions of a CO_2_ incubator (37 °C, 5% CO_2_) for 24, 48 and 72 h.

### 2.3. Immunocytochemistry

Cells were fixed on coverslips in 4% paraformaldehyde in PBS (pH 7.4) for 20 min at room temperature followed by two rinses with PBS and permeabilization in 0.3% Triton-X100 (in PBS, pH 7.4) supplemented with 5% goat serum to block non-specific antibody binding for 60 min. Cells were then incubated overnight at 4 °C with primary rabbit monoclonal antibodies against γH2AX (phospho S139) (dilution 1:800, clone EP854(2)Y, Abcam, Waltham, MA, USA), or primary mouse monoclonal antibodies against 53BP1 (dilution 1:400 clone BP13, Merck-Millipore, Burlington, VT, USA), or primary mouse monoclonal antibodies against phosphorylated ATM (phospho S1981) protein (dilution 1:200, clone 10H11.E12, Abcam, Waltham, MA, USA), or primary mouse monoclonal antibodies against phospho-p53 (Ser15) (16G8) (dilution 1:400, Cell signaling, Danvers, MA, USA), or primary mouse monoclonal antibodies against Ki-67 protein (dilution 1:400, clone Ki-S5, Merck-Millipore, Burlington, VT, USA) or primary rabbit polyclonal antibody against LC3B-I/II proteins (dilution 1:400, cat. # ABC929, Merck-Millipore, Burlington, VT, USA) or primary rabbit monoclonal antibodies against cleaved caspase-3 (dilution 1:400, cat. # 9664, Cell signaling, Danvers, MA, USA), which were diluted in PBS with 1% bovine serum albumin (BSA). After several rinses with PBS, cells were incubated for 1 h with secondary antibodies IgG (H + L) goat anti-mouse (Alexa Fluor 488 conjugated, 1:1600; Abcam, Waltham, MA, USA) or goat anti-rabbit IgG H&L (Alexa Fluor^®^ 555) goat anti-rabbit dilution 1:1600; Abcam, Waltham, MA, USA) and diluted in PBS (pH 7.4) with 1% BSA. Coverslips were then rinsed several times with PBS and mounted on microscope slides with ProLong Gold medium (Life Technologies, Carlsbad, SA, USA) with DAPI for DNA counterstaining. Cells were viewed and imaged using Nikon Eclipse Ni-U microscope (Nikon, Tokyo, Japan) equipped with a high-definition camera ProgRes MFcool (Jenoptik AG, Jena, Germany). We used filter sets UV-2E/C (340–380 nm excitation and 435–485 nm emission), B-2E/C (465–495 nm excitation and 515–555 nm emission) and Y-2E/C (540–580 nm excitation and 600–660 nm emission). A total of 300–400 cells were imaged for each data point. Foci were enumerated by manual scoring and using DARFI software (http://github.com/varnivey/darfi; accessed on 19 September 2016). Representative raw microphotographs of immunocytochemically stained control and 10 Gy irradiated fibroblasts are presented in Figure S1. The proportions of Ki67 negative and LC3-II or caspase-3 positive cells were counted manually (300 cells per data point).

### 2.4. Analysis of Senescence Associated β-galactosidase Positive Cells

To quantify the proportion of senescence associated β-galactosidase positive (SA-β-gal+) cells, the commercial kit “Cellular Senescence Assay” (EMD Millipore, Burlington, VT, USA, Catalog Number: KAA002) was used. The cells were stained according to supplemented manufacturer protocol with the following modification: at the final PBS washing step, the cell nuclei were stained with 1 μg/mL Hoechst 33342 (Molecular Probes, Eugene, OR, USA). Such modification significantly improves the quality of counting of β-galactosidase negative cells [24,25]. The stained cells were visualized using Excitation/Emission Interference Filters (CKX-U: 340–380 nm/435–485 nm) on the inverted fluorescent microscope Olympus CKX 41 SF (Olympus, Tokyo, Japan) equipped with Infinity 3-1 (Lumenera Copr., Ottawa, Canada) camera and 20× objective. The proportions of SA-β-gal+ cells were counted manually (300 cells per each data point).

### 2.5. Statistical Analysis

Statistical and mathematical analyses of the data were conducted using the Statistica 8.0 software (StatSoft). The results are presented as means of three independent experiments ± standard error (SE). Statistical significance was tested using the Student’s *t*-test and Mann–Whitney U Test.

## 3. Results

### 3.1. Dose and Post-Irradiation Tim- Dependent Changes in the Residual Foci of DNA Damage Response Proteins

**Figure 1:**
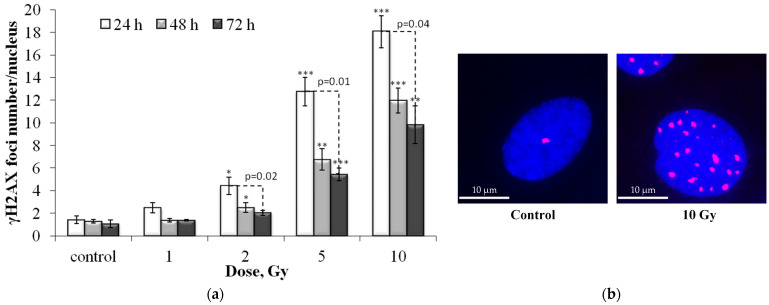
(**a**) Dose–dependent changes in the number of residual γH2AX foci in human fibroblasts 24 h, 48 h and 72 h after irradiation. Data are means ± SE of three independent experiments. * *p* < 0.05; ** *p* < 0.01 and *** *p* < 0.001—compared with the corresponding time of control; (**b**) Representative microphotographs of immunocytochemically stained control and 10 Gy irradiated cells (24 h) showing nucleus (blue) and foci (red).

Post-irradiation changes in the number of residual γH2AX foci in fibroblasts are depicted in Figure 1. It was shown that the number of residual γH2AX foci in irradiated cells depends linearly on the absorbed dose of X-ray radiation. The dose–response relationships are approximated by linear equations, where y is the number of γH2AX foci, x is the dose in Gy: y = 1.76x + 1.50 (R^2^ = 0.96), y = 1.13x + 0.71 (R^2^ = 0.99) and y = 0.92x + 0.65 (R^2^ = 0.99), 24, 48 and 72 h after irradiation, respectively (Figure S2a). The slope shows the change in the number of foci per unit of dose. The most significant decrease (~1.6 times) in the quantitative yield of residual γH2AX foci occurs in the period from 24 to 48 h after irradiation. After that, from 48 to 72 h, the quantitative yield of residual foci decreases less significantly (~1.2 times). In the period from 24 to 72 h, the quantitative yield of residual foci decreases by ~1.9 times.

**Figure 2:**
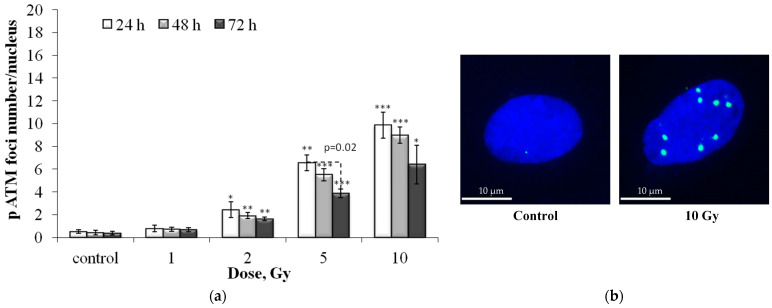
(**a**) Dose-dependent changes in the number of residual pATM foci in human fibroblasts 24 h, 48 h and 72 h after irradiation. Data are means ± SE of three independent experiments. * *p* < 0.05, ** *p* < 0.01 and *** *p* < 0.001—compared with the corresponding time of control; (**b**) Representative microphotographs of immunocytochemically stained control and 10 Gy irradiated cells (24 h) showing nucleus (blue) and foci (green).

Figure 2 presents post-radiation changes in the number of residual pATM foci. The dose–response relationships are approximated by linear equations, where y is the number of pATM foci, x is the dose in Gy: y = 0.99x + 0.47 (R^2^ = 0.97), y = 0.90x + 0.27 (R^2^ = 0.98) and y = 0.62x + 0.36 (R^2^ = 0.99), 24, 48 and 72 h after irradiation, respectively (Figure S2b). It can be seen that in the period from 24 to 72 h after irradiation, there is a significant (~1.6 times) decrease in the quantitative yield of residual foci.

**Figure 3:**
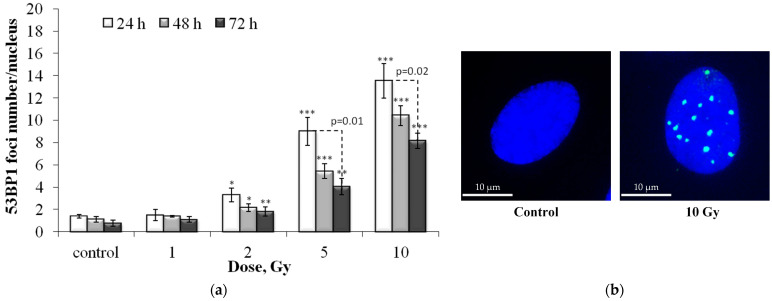
(**a**) Dose-dependent changes in the number of residual 53BP1 foci in human fibroblasts 24 h, 48 h and 72 h after irradiation. Data are means ± SE of three independent experiments. * *p* < 0.05, ** *p* < 0.01 and *** *p* < 0.001—compared with the corresponding time of control; (**b**) Representative microphotographs of immunocytochemically stained control and 10 Gy irradiated cells (24 h) showing nucleus (blue) and foci (green).

Figure 3 presents post-radiation changes in the number of residual 53BP1 foci. The dose–response relationships are approximated by linear equations, where y is the number of 53BP1 foci, x is the dose in Gy: y = 1.31x + 1.05 (R^2^ = 0.97), y = 0.97x + 0.61 (R^2^ = 0.99) and y = 0.76x + 0.45 (R^2^ = 0.99), 24, 48 and 72 h after irradiation, respectively (Figure S2c). In the period from 24 to 72 h, a decrease in the number of residual foci by ~1.7 times is noted.

**Figure 4:**
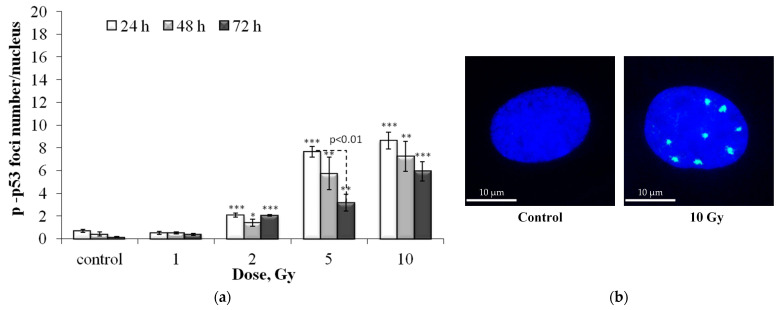
(**a**) Dose-dependent changes in the number of residual p-p53 foci in human fibroblasts 24 h, 48 h and 72 h after irradiation. Data are means ± SE of three independent experiments. * *p* < 0.05, ** *p* < 0.01 and *** *p* < 0.001—compared with the corresponding time of control; (**b**) Representative microphotographs of immunocytochemically stained control and 10 Gy irradiated cells (24 h) showing nucleus (blue) and foci (green).

Figure 4 presents post-radiation changes in the number of p-p53 residual foci.

The dose–response relationships are approximated by linear equations, where y is the number of p-p53 foci, x is the dose in Gy: y = 0.90x + 0.67 (R^2^ = 0.86), y = 0.76x + 0.34 (R^2^ = 0.91) and y = 0.58x + 0.27 (R^2^ = 0.97), 24, 48 and 72 h after irradiation, respectively (Figure S2d). As in the case of residual γH2AX, pATM, and 53BP1 foci, with increasing time after irradiation, the quantitative yield of p-p53 foci decreases (from 24 to 72 ~ 1.6 times).

Table A1 shows the quantitative yield of residual foci per unit of absorbed dose (foci/cell/Gy) obtained using regression analysis of dose curves.

### 3.2. Colocalization of pATM, 53BP1 and p-p53 Foci with γH2AX Foci

**Figure 5:**
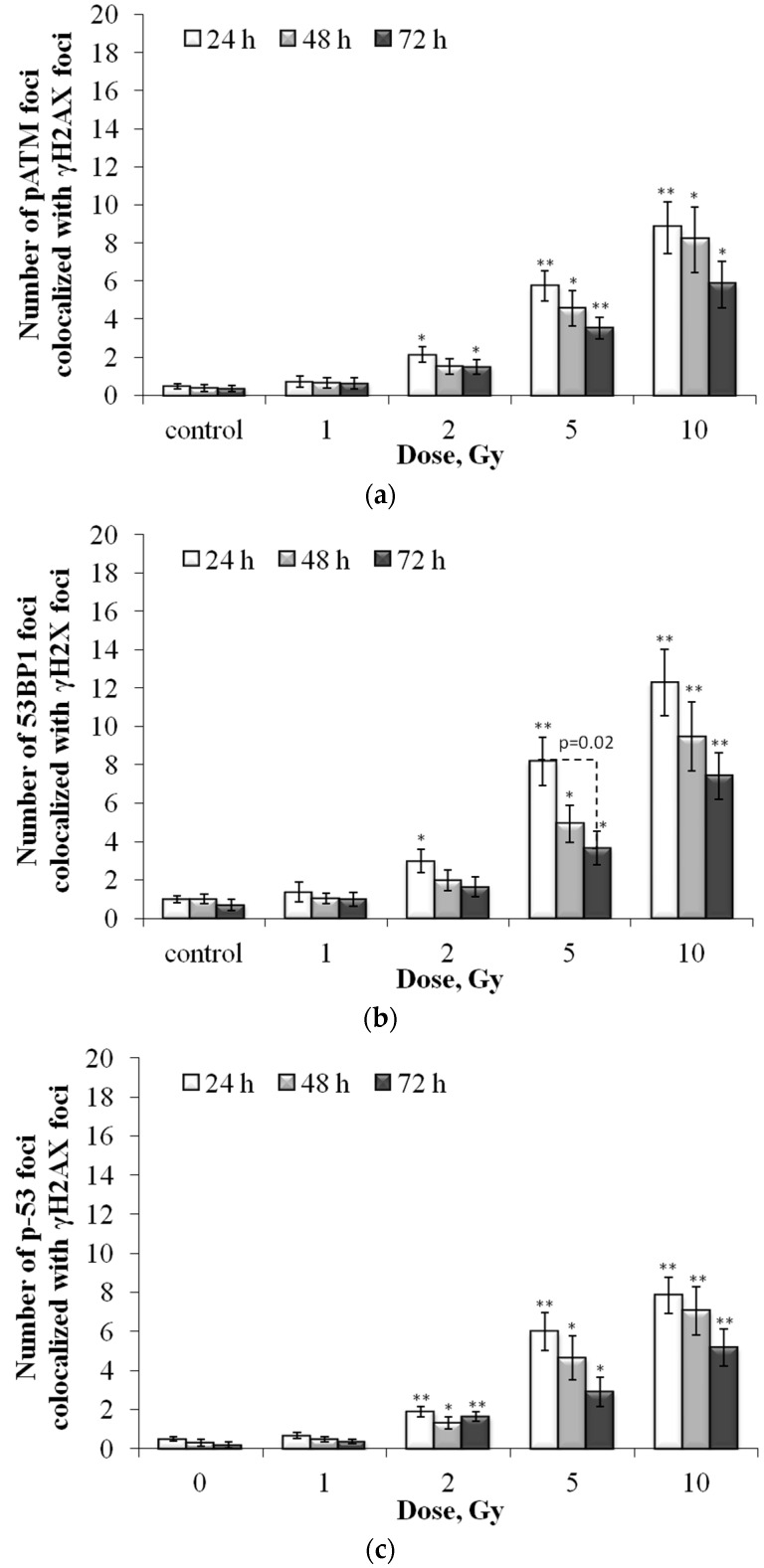
Dose-dependent changes in the number of pATM (**a**), 53BP1 (**b**) and p-p53 (**c**) foci colocalized with γH2AX foci in human fibroblasts 24 h, 48 h and 72 h after irradiation. Data are means ± SE of three independent experiments. * *p* < 0.05 and ** *p* < 0.01—compared with the corresponding time of control.

Dose-dependent changes in the number of pATM, 53BP1 and p-p53 foci colocalized with γH2AX foci are shown in Figure 5.

The obtained “dose-effect” dependences are close to those obtained by counting foci without taking into account co-localization with γH2AX. However, the quantitative yield of colocalized foci was somewhat lower (Table A2). Furthermore, 24 h after irradiation, ~65–70% of γH2AX foci were colocalized with 53BP1 foci, whereas after 48 and 72 h it was about 70–80%.

The pattern of changes in the proportion of colocalized γH2AX foci with pATM and p-p53 foci was similar. 24 h after irradiation only 45–50% of γH2AX foci were colocalized with pATM or p-p53, but with a decrease in proliferative activity 48 and 72 h after irradiation, up to 60–70% of γH2AX foci were colocalized with pATM or p-p53 foci.

### 3.3. Dose and Post-Irradiation Time-Dependent Changes in the Senescent Cell Proportion

**Figure 6:**
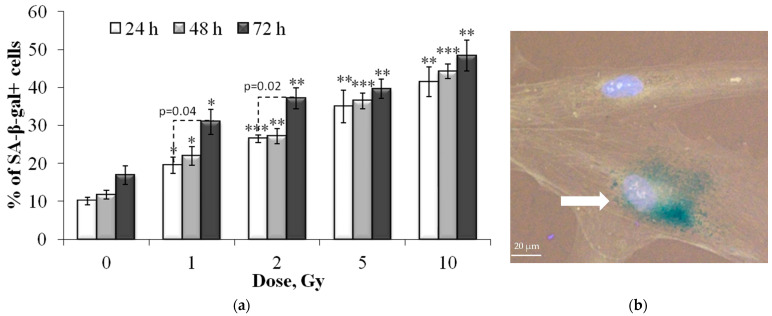
(**a**) Dose-dependent changes in the senescent cell (SA-β-gal+) proportion in human fibroblasts 24 h, 48 h and 72 h after irradiation. Data are means ± SE of three independent experiments. * *p* < 0.05, ** *p* < 0.01 and *** *p* < 0.001—compared with the corresponding time of control; (**b**) Representative image of the SA-β-gal positive cell (marked with arrow—cytoplasm colored in dark green-blue). Nuclei are counterstained with Hoechst 33342 (light blue).

The senescence associated β-galactosidase (SA-β-gal) staining analysis was performed (Figure 6) to evaluate the senescent cell proportion changes in irradiated cells. This enzyme is commonly used as a marker of cellular senescence since its expression is substantially elevated in senescent cells [24,26].

It was shown that exposure to X-ray radiation at doses of 1–10 Gy leads to a dose-dependent increase in the proportion of senescent SA-β-gal positive cells (Figure 6) as early as 24 h after exposure. With an increase in the time of post-radiation incubation of cells up to 72 h, the proportion of senescent fibroblasts also increased. However, a statistically significant increase was noted only for the cells irradiated at doses of 1 and 2 Gy (Figure 6). Dose–response relationships 24 and 48 h after irradiation can be approximated by linear equations, where y is the proportion of SA-β-gal positive cells, x is the dose in Gy: y = 2.84x + 16.32 (R^2^ = 0.85) and y = 2.94x + 17.75 (R^2^ = 0.88), respectively. After 72 h, a linear increase in the proportion of SA-β-gal positive cells was noted at doses up to 2 Gy, after which a “saturation” effect was observed (Figure 6).

### 3.4. Dose and Post-Irradiation Time–Dependent Changes in the Ki-67 Negative Cell Fraction and Differential Scoring of γH2AX Foci in the Ki-67 Negative Cells

**Figure 7:**
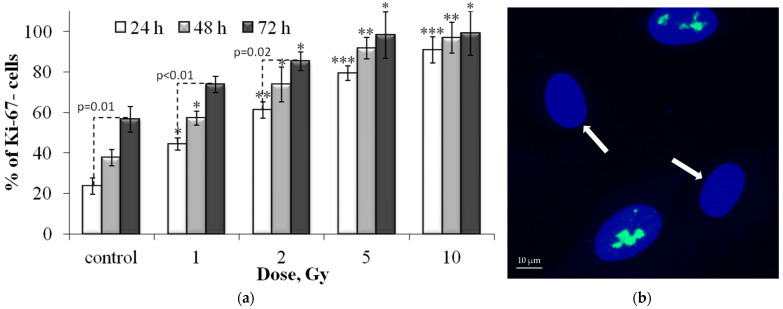
(**a**) Dose-dependent changes in the Ki-67 negative (Ki-67-) cell fraction in human fibroblasts 24 h, 48 h and 72 h after irradiation. Data are means ± SE of three independent experiments. * *p* < 0.05, ** *p* < 0.01 and *** *p* < 0.001—compared with the corresponding time of control; (**b**) Representative microphotograph of the immunocytochemically labeled cells with the Ki-67 antibodies (green) (Ki-67- cells are marked with the arrows). Nuclei are counterstained with DAPI (blue).

Figure 7 shows the results of the proportion of Ki-67 negative cells in populations of control and irradiated fibroblasts. The Ki-67 protein is expressed in cells during interphase (with a maximum in the S and G2 phases) and mitosis (M) and is practically absent in resting and senescent cells [27,28]. Ki-67 has been shown to be involved in ribosome biogenesis, heterochromatin organization and mitotic chromosome segregation [28,29].

Analysis of changes in the proportion of Ki-67-negative cells (Ki-67-) in the control group showed that with an increase in the incubation time, their number increases due to the growth of the cell population and contact inhibition of proliferation (Figure 7). Notably, the cells were seeded in Petri dishes 24 h before irradiation, that is, 48, 72, and 96 h passed for the control cell populations. By 96 h of incubation, cells stop exponential growth and stop dividing. Irradiation of cells causes a dose-dependent decrease in proliferative activity (Figure 7) due to the arrest of the cell cycle and loss of the ability to divide due to radiation-induced senescence. The most pronounced effect was observed 72 h after irradiation (Figure 7). A linear increase in the proportion of Ki-67 negative cells after irradiation was noted at doses up to 5 Gy at 24 and 48 h and up to 2 Gy at 72 h, after which a “plateau” effect was observed (Figure 7). In cells irradiated at a dose of 10 Gy, there is practically no proliferative activity. In general, the results obtained correlate well with changes in the proportion of SA-β-gal positive cells in the populations of irradiated fibroblasts.

**Figure 8:**
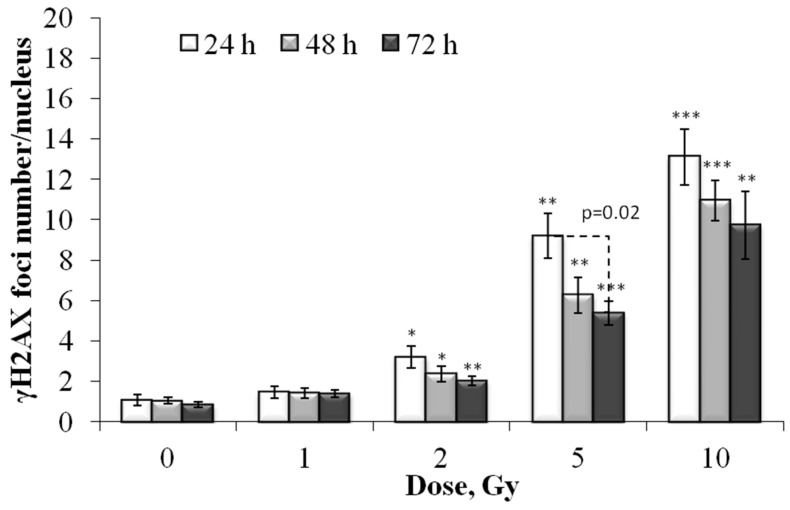
Dose-dependent changes in the number of residual γH2AX foci in the Ki-67 negative human fibroblasts 24 h, 48 h and 72 h after irradiation. Data are means ± SE of three independent experiments. * *p* < 0.05, ** *p* < 0.01 and *** *p* < 0.001—compared with the corresponding time of control.

It was interesting to analyze γH2AX foci only in resting (Ki-67 negative) fibroblasts. The results are presented in Figure 8. Mathematical analysis of the obtained results showed that the dose–response relationships are approximated by linear equations, where y is the number of γH2AX foci, x is the dose in Gy: y = 1.29x + 0.96 (R2 = 0.96), y= 1.04x + 0.66 (R2 = 0.99) and y = 0.92x + 0.57 (R2 = 0.99), 24, 48 and 72 h after irradiation, respectively. Thus, the quantitative yield of γH2AX foci in resting cells 24 h after irradiation was significantly lower than in the total cell population (1.29 and 1.76 foci/cell/Gy, respectively). With a decrease in proliferative activity by 72 h after irradiation, when most of the cells, especially those irradiated at doses of 5 and 10 Gy, were dormant, the numerical values of the quantitative yield no longer differed.

### 3.5. Dose and Post-Irradiation Time-Dependent Changes in the LC3-II Positive Cell Fraction

LC3 is a mammalian homolog of the yeast ATG8 protein, a ubiquitin-like protein that becomes lipidated and tightly associated with the autophagosomal membranes [30]. During autophagy, the cytosolic form of LC3 (LC3-I) is conjugated with phosphatidylethanolamine to form the LC3-phosphatidylethanolamine conjugate (LC3-II), which is incorporated into the membranes of the resulting autophagosomes [31]. When immunocytochemically stained for LC3-II, autophagosomes are visualized as the punctate (granular/vesicular) compartments of the cytoplasm, which makes it easy to differentiate autophagic cells.

**Figure 9:**
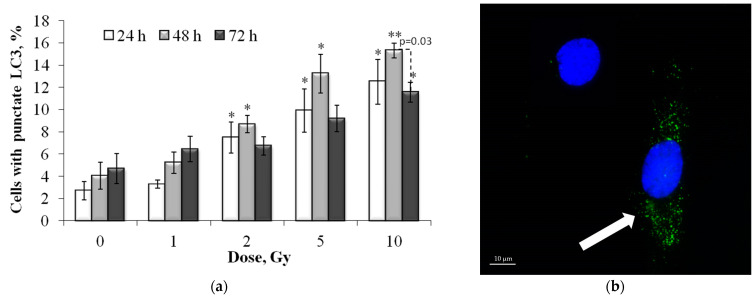
(**a**) Dose-dependent changes in the LC3-II positive cell fraction in human fibroblasts 24 h, 48 h and 72 h after irradiation. Data are means ± SE of three independent experiments. * *p* < 0.05 and ** *p* < 0.01—compared with the corresponding time of control; (**b**) Representative microphotograph of the immunocytochemically labeled cells with LC3-II (LC3-II positive cell with punctate LC3-II (green) is marked with the arrow). Nuclei are counterstained with DAPI (blue).

The results of the analysis of changes in the proportion of autophagic (LC3-II positive) cells in irradiated cells are shown in Figure 9. It was shown that 24 and 48 h after irradiation there was a linear increase in the proportion of autophagic cells depending on the absorbed dose, with a maximum at 48 h. The dose–response relationships are approximated by linear equations, where y is the percent of the LC3-II positive cells, x is the dose in Gy: y = 0.98x + 3.66 (R2 = 0.87), y = 1.14x + 5.18 (R2 = 0.88), 24 and 48 h after irradiation, respectively. Following 72 h after irradiation, the proportion of autophagic cells decreases compared to the values at 48 h, and statistically significant differences compared to the control are noted only after irradiation at a dose of 10 Gy (Figure 9).

### 3.6. Dose and Post-Irradiation Time-Dependent Changes in the Caspase-3 Positive Cell Fraction

Effector caspase-3, belonging to the family of cysteine-aspartate proteases (caspase), is widely known primarily due to its important role in the process of cell death through apoptosis [32]. However, the functions of caspase-3 are much wider, in particular, it is important for the regulation of autophagy processes [33] and the balance between the processes of cell death and aging [34]. According to recent data, caspase-3 cleavage of specific target proteins also regulates cell cycle progression, differentiation, and tumorigenesis [35]. Therefore, it was important to study changes in the proportion of caspase-3 positive cells in populations of irradiated fibroblasts.

**Figure 10:**
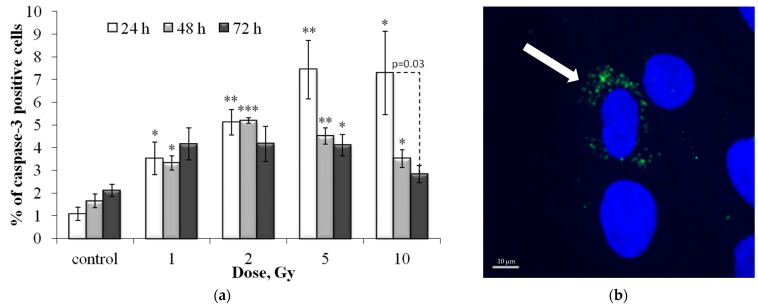
(**a**) Dose-dependent changes in the caspase-3 positive cell fraction in human fibroblasts 24 h, 48 h and 72 h after irradiation. Data are means ± SE of three independent experiments. * *p* < 0.05 and ** *p* < 0.01, *** *p* < 0.001—compared with the corresponding time of control; (**b**) Representative microphotograph of the immunocytochemically labeled cells with caspase-3 (caspase-3 positive cell with punctate caspase-3 (green) is marked with the arrow). Nuclei are counterstained with DAPI (blue).

As can be seen from the results presented in Figure 10, a dose-dependent increase in caspase-3 positive cells was noted only 24 h after irradiation. At 48 and 72 h after irradiation at doses of 1 and 2 Gy, the proportion of caspase-3 positive cells changed insignificantly, while after irradiation at doses of 5 and 10 Gy, a pronounced decrease in the proportion of caspase-3 positive cells was noted (Figure 10). That is, at these time points, the change in the proportion of caspase-3 positive cells depended non-linearly on the radiation dose.

### 3.7. Correlation and Clustering Analysis

Table A3 presents the results of the correlation analysis between all the studied indicators.

It was shown that 24 h after irradiation, a statistically significant positive correlation was noted for almost all the parameters studied, with the exception of the correlation between the number of γH2AX, 53BP1 foci and pATM shares of caspase-3 positive cells (Table A3). It is interesting to note that there is a significant correlation between the number of p-p53 effector protein foci and the proportion of caspase-3 positive cells.

Further, 48 h after irradiation, the correlation matrix changed significantly: (1) there was no significant correlation between the proportion of caspase-3 positive cells and other studied parameters; (2) there was no significant correlation between the number of γH2AX/53BP1 foci and the proportion of resting Ki-67 negative cells (Table A3).

Even more significant changes in the correlation matrix were observed 72 h after irradiation: (1) there was still no significant correlation between the proportion of caspase-3 positive cells and other studied parameters, moreover, the coefficients decreased even more and for DDR foci there was even a tendency to a negative correlation; (2) the proportion of Ki-67 negative cells significantly correlated only with the proportion of LC3-II and SA-β-gal positive cells; (3) the proportion of senescent SA-β-gal positive cells correlated only with the number of p-p53 foci, as well as the proportion of LC3-II positive and the proportion of Ki-67 negative cells (Table A3).

**Figure 11:**
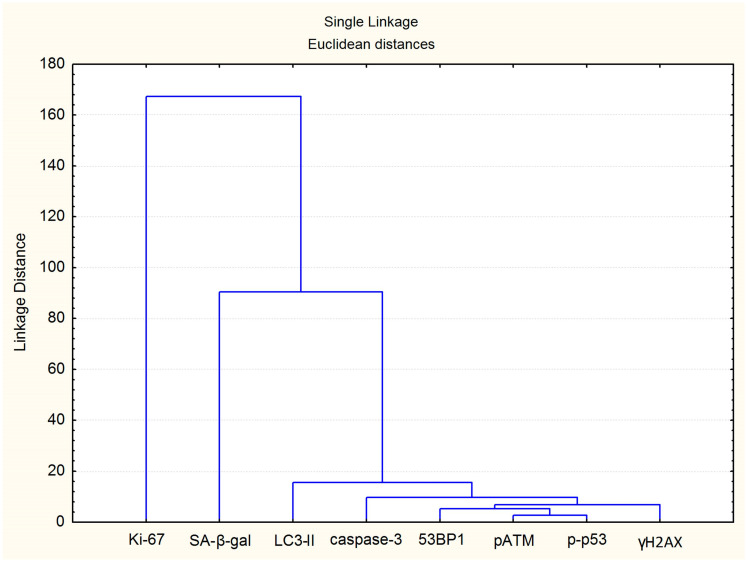
Clustering tree for the investigated endpoints.

To understand the overall picture of the relationship between changes in the studied indicators, it seemed interesting to conduct a cluster analysis with the construction of a hierarchical cluster tree. It has been shown that the most similar are changes in the number of foci of DRR proteins with the following hierarchy in order of increasing linkage distance: (1) pATM and p-p53; (2) pATM, p-p53 and 53BP1; (3) pATM, p-p53, 53BP1 and γH2AX (Figure 11). The following branches were identified at the following levels: (4) foci of DDR and caspase-3 proteins; (5) DDR proteins, caspase 3 and LC3-II; (6) foci of DDR, caspase 3 and LC3-II proteins; (7) foci of DDR, caspase 3, LC3-II, and SA-β-gal proteins (Figure 11). The resulting cluster tree visually demonstrates the development of a cell response to irradiation 24–72 h after fibroblast irradiation at doses of 1–10 Gy: unrepaired and complex DNA damage induces DDR, which primarily induces cell death through the mechanisms of apoptosis and autophagy with a progressive switch to a senescent state.

## 4. Discussion

In the course of our work, for the first time, a simultaneous study of dose–response dependences and post-radiation changes in the residual foci of proteins, representing all the main functional classes of DDR proteins (sensors, transducers, mediators and effectors), was carried out in X-ray irradiated human fibroblasts. It was shown that the highest quantitative yield is observed for the γH2AX protein foci. γH2AX is an initial DSB sensor for subsequent accumulation and post-translational modification of signaling and repair proteins [36,37]. The association between γH2AX and the mediator of DNA damage checkpoint protein 1 (MDC1) is one of the first phases during which the DSB is arranged for DNA damage signaling and repair [38,39]. To this initial step, several other signaling and repair proteins, such as 53BP1 and the breast cancer gene protein 1 (BRCA1), accumulate at DSBs with the intervention of γH2AX [39,40]. Transducer protein ATM, autophosphorylated immediately upon DSB formation (pATM), is the main kinase phosphorylating H2AX [41]. In addition to ATM, this histone is phosphorylated by other kinases of the phosphatidylinositol 3-kinases ataxia telangiectasia and Rad3-related (ATR) and DNA-dependent protein kinase catalytic subunit (DNA-PKcs) family [42,43]. However, unlike the ATM and DNA-PKcs kinases, the ATR kinase is activated in response to the formation of not only true DSBs, but upon the appearance of single-stranded DNA regions, for example, the formation of DSBs with sticky ends as a result of the collapse of the replication fork [44]. Unlike DNA DSBs directly induced by radiation, S-phase replication errors typically result in single-stranded DNA lesions and the formed single-ended DSBs are processed by homologous recombination [45,46]. The quantitative yield of radiation-induced residual pATM foci 24 h after irradiation was approximately 1.8 times lower than the quantitative yield of residual γH2AX foci. It can be assumed that some of the observed γH2AX foci at this time point are the repair sites for single-ended DSBs resulting from the collapse of replication forks. This assumption is confirmed by the fact that that the quantitative yield of γH2AX foci in Ki-67 negative resting cells 24 h after irradiation was significantly lower than in the total cell population (1.29 and 1.76 foci/cell/Gy, respectively).

Another important protein involved in DDR is the mediator protein 53BP1. Although 53BP1 was discovered and named based on its interaction with p53, 53BP1 has been most thoroughly characterized in terms of its role at broken DNA ends where it recruits effector proteins to mediate DSB repair [47]. The C-terminal region of 53BP1 contains two carboxyterminal (BRCT) domains of BRCA1, which interact with p53 and γH2AX, which is important for DSB repair in heterochromatin [48]. 53BP1, together with the RAP1-interacting factor (RIF1) and the Pax2 transactivation domain-interacting protein, has been shown to promote NHEJ selection and to inhibit HR [49,50]. The quantitative yield of residual 53BP1 foci was lower than that of γH2AX foci (~1.3–1.4 times after 24 h and ~1.1–1.2 times 48 and 72 h after irradiation). By, 24 h after irradiation, ~70–75% of γH2AX foci were colocalized with 53BP1 foci, whereas after 48 and 72 h it was about 80–90%.

The next studied protein was the effector protein p53 phosphorylated at Serine 15. Serine 15 is the primary target of the DDR on the p53 protein and is phosphorylated by both the ATM and ATR protein kinases [51,52]. Unlike p53 phosphorylation at Serine 46, which plays an important role in apoptosis, p53 phosphorylation at Serine 15 leads to cell cycle arrest. [53] ATM-mediated phosphorylation of p53 and checkpoint kinase 2 (CHK2) leads to a series of G1 and G2 cell cycle checkpoints that act together in preventing genomic instability following DNA damage [16,54]. It was shown that the quantitative yield of residual foci of p-p53 and pATM as well as the percent of colocalization with γH2AX foci are similar.

In general, according to the quantitative yield of residual foci, the studied proteins can be arranged in descending order γH2AX > 53BP1 > pATM ≥ p-p53. With an increase in time after irradiation from 24 to 72 h, the number of residual foci of all studied proteins decreases. The decrease in the number of residual foci can be explained by several parallel processes: the elimination of highly damaged cells through the mechanisms of apoptosis, autophagy, etc.; the completion of the DNA repair process; and, finally, a decrease in the proliferative activity of cells, accompanied by a decrease in the amount of replicative DNA damage.

X-ray exposure also led to a dose-dependent increase in the proportion of caspase-3, LC3-II, SA-β-gal positive and Ki-67 negative fibroblasts 24 h after irradiation at doses of 1-10 Gy. By 72 h after irradiation, the proportion of dying (caspase-3 and LC3-II) positive cells decreases, while the proportion of senescent cells, on the contrary, increases. Irradiated cells, in response to the formation of DNA DSBs, activate cell cycle control points via the ATM/ATR signaling pathway, causing a delay or arrest of the cell cycle at certain phases, allowing time for DNA repair. Activated forms of ATM/ATR regulate the activation of cell cycle checkpoints associated with cell death and senescence, mainly through p53, CHK1 and CHK2 with the participation of p21, p16 and Rb [55]. Interestingly, the largest proportion of caspase-3 positive cells was noted 24 h after irradiation, and the proportion of LC3-II positive cells after 48 h. That is, a decrease in caspase-3 positive cells after 48 h is accompanied by an increase in the proportion of LC3-II positive cells, which suggests that damaged cells are quickly eliminated by apoptosis, while autophagic cell death progresses slower as the accumulated damages cannot be cleared by the cell. Also noteworthy is the fact that 48–72 h after irradiation, the proportion of caspase-3 cells did not correlate with any of the studied parameters (Table A2).

**Figure 12:**
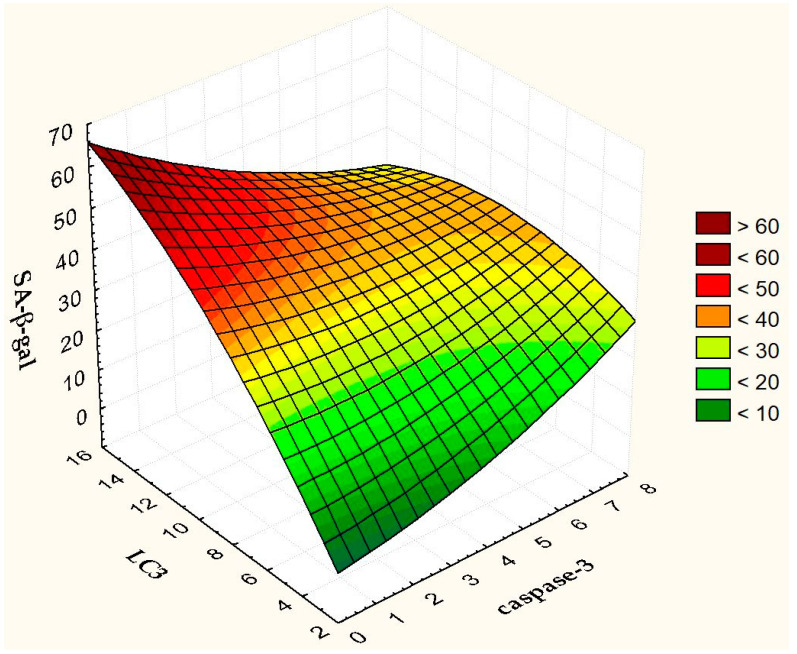
3D Surface Plot of SA-β-gal against caspase-3 and LC3.

Figure 12 presents a 3D Surface Plot to show how the proportion of SA-β-gal+ cells varies with changes in the proportion of caspase-3 and LC3 positive cells. It is noteworthy that senescence and autophagy act as partners, reinforcing each other and acting in opposition to apoptosis (Figure 12).

Autophagy and apoptosis are known to cross-regulate each other through a complex network of relationships that also includes interactions between autophagy-related proteins and caspases [33]. A decrease in the activity of caspase-3, which is involved in the cleavage of various autophagy-related proteins (Atgs) [56], 48 h after irradiation, apparently promotes the process of autophagy. It is interesting that replicatively senescent fibroblasts are resistant to apoptotic death, associated with a lack of caspase-3 [57]. We have previously shown that the transcriptome of replicatively aged fibroblasts is similar to the transcriptome of the cells irradiated with 2 Gy [58]. Recent studies have shown that autophagy may conversely promote cellular senescence by facilitating the synthesis of secretory proteins associated with aging [59]. Autophagy is activated during senescence and its activation is correlated with negative feedback in the PI3K-mammalian target of rapamycin (mTOR) pathway [60]. The proportion of autophagic cells correlated with the proportion of senescent cells up to 72 h after irradiation. It is known that cell death, as well as senescence, are mechanisms of protection of normal cells from oncotransformation [61,62]. In the first case, severely damaged, potentially dangerous cells are eliminated, and in the second, they lose their ability to divide. Here natural questions arise. Why not eliminate all severely damaged cells? After all, senescent cells secrete senescence-associated phenotype factors that can amplify cellular senescence, alter the microenvironments, and even provoke oncogenesis [63,64]. Is there any biological sense in maintaining the viability of senescent cells? Recently, on the contrary, evidence has begun to appear that the use of senolytics (substances that remove senescent cells) or senostatics (substances that inhibit paracrine signaling of senescent cells) can reduce damage to normal tissues [63]. However, the question remains open and requires careful study.

## 5. Conclusions

In conclusion, the studies emphasize the important role of DSBs that are difficult to repair in initiating the process of radiation-induced cell death and senescence. For the first time in one work, a simultaneous study of the association of changes in the residual foci of key DDR proteins, the proportion of caspase-3 positive, autophagic and senescent cells was carried out 24–72 h after fibroblast irradiation at doses of 1–10 Gy. It was shown that with an increase in time after irradiation from 24 h to 72 h, the number of residual foci and the proportion of caspase-3 positive cells decrease, while the proportion of senescent cells, on the contrary, increases. The highest number of autophagic cells was noted 48 h after irradiation. According to the quantitative yield of residual foci, the studied proteins can be arranged in descending order γH2AX > 53BP1 > pATM ≥ p-p53. Of particular interest are the results of the analysis of p-p53 (Serine 15) foci obtained by us for the first time on irradiated fibroblasts. This is the only DDR protein studied, the number of foci of which significantly correlated with the proportion of caspase-3 positive cells 24 h after irradiation, and 72 h after irradiation with the proportion of SA-β-gal positive cells. That is, p-p53 (Serine 15) residual foci potentially have the greatest prognostic value in assessing the fate of irradiated cells. In general, the results obtained provide important information for understanding the dynamics of the development of a dose-dependent cellular response in irradiated fibroblast populations 24–72 hours after irradiation. This study highlights the pathways of radiation-induced damage of normal tissue cells located in the path of IR. A thorough understanding of these mechanisms is required to identify molecular targets to develop radioprotection strategies for normal tissue cells. The quantitative “dose-effect” patterns obtained by us are also extremely important for the further development and improvement of radiation biodosimetry.

## Data Availability

Not applicable.

## Supplementary Materials

The following supporting information can be downloaded at: https://www.mdpi.com/article/10.3390/cells12081209/s1, Figure S1: Representative raw microphotographs of immunocytochemically stained control and 10 Gy irradiated fibroblasts. Figure S2: The dose-response curves and mathematical fits for the residual foci in human fibroblasts 24 h, 48 h and 72 h after irradiation: γH2AX (a), pATM (b), 53BP1 (c) and p-p53 (d) foci. Data are means ± SE of three independent experiments.

## Appendix A

**Table A1:** The yield of residual foci of DNA damage response proteins per unit of absorbed dose (foci/cell/Gy) 24, 48 and 72 h after X-ray exposure.

Protein	Time after X-ray Exposure, h		
	24	48	72
γH2AX	1.76 ± 0.20	1.13 ± 0.07	0.92 ± 0.05
pATM	0.99 ± 0.10	0.90 ± 0.07	0.62 ± 0.04
53BP1	1.31 ± 0.13	0.97 ± 0.05	0.76 ± 0.03
p-p53	0.90 ± 0.21	0.76 ± 0.14	0.58 ± 0.06

**Table A2:** The yield of pATM, 53BP1 and p-p53 foci colocalized with γH2AX foci per unit of absorbed dose (foci/cell/Gy) 24, 48 and 72 h after X-ray exposure.

Protein	Time after X-ray Exposure, h		
	24	48	72
pATM	0.88 ± 0.08	0.82 ± 0.04	0.57 ± 0.04
53BP1	1.20 ± 0.12	0.89 ± 0.05	0.69 ± 0.03
p-p53	0.80 ± 0.13	0.73 ± 0.08	0.51 ± 0.04

**Table A3:** Correlation matrices for the investigated endpoints at 24, 48 and 72 h after X-ray exposure.

	γH2AX	53BP1	pATM	p-p53	caspase-3	LC3-II	SA-β-gal	Ki-67
24 h								
**γH2AX**	-	0.998 *	0.998 *	0.983 *	0.877	0.952 *	0.939 *	0.928 *
**53BP1**	0.998 *	-	0.999 *	0.977 *	0.853	0.950 *	0.926 *	0.913 *
**pATM**	0.998 *	0.999 *	-	0.978 *	0.872	0.963 *	0.941 *	0.929 *
**p-p53**	0.983 *	0.977 *	0.978 *	-	0.889 *	0.944 *	0.920 *	0.914 *
**caspase-3**	0.877	0.853	0.872	0.889 *	-	0.934 *	0.976 *	0.984 *
**LC3-II**	0.952 *	0.950 *	0.963 *	0.944 *	0.934 *	-	0.974 *	0.972 *
**SA-β-gal**	0.939 *	0.926 *	0.941 *	0.920 *	0.976 *	0.974 *	-	0.999 *
**Ki-67**	0.928 *	0.913 *	0.929 *	0.914 *	0.984 *	0.972 *	0.999 *	-
**48 h**								
**γH2AX**	-	0.998 *	0.996 *	0.970 *	0.218	0.938 *	0.916 *	0.851
**53BP1**	0.998 *	-	0.991 *	0.956 *	0.200	0.923 *	0.909 *	0.837
**pATM**	0.996 *	0.991 *	-	0.984 *	0.286	0.964 *	0.940 *	0.889 *
**p-p53**	0.970 *	0.956 *	0.984 *	-	0.312	0.972 *	0.932 *	0.901 *
**caspase-3**	0.218	0.200	0.286	0.312	-	0.513	0.569	0.682
**LC3-II**	0.938 *	0.923 *	0.964 *	0.972 *	0.513	-	0.976 *	0.970 *
**SA-β-gal**	0.916 *	0.909 *	0.940 *	0.932 *	0.569	0.976 *	-	0.984 *
**Ki-67**	0.851	0.837	0.889 *	0.901 *	0.682	0.970 *	0.984 *	-
**72 h**								
**γH2AX**	-	0.997 *	0.994 *	0.974 *	−0.143	0.970 *	0.827	0.799
**53BP1**	0.997 *	-	0.990 *	0.979 *	−0.150	0.965 *	0.835	0.790
**pATM**	0.994 *	0.990 *	-	0.988 *	−0.063	0.981 *	0.866	0.854
**p-p53**	0.974 *	0.979 *	0.988 *	-	−0.036	0.966 *	0.895 *	0.864
**caspase-3**	−0.143	−0.150	−0.063	−0.036	-	0.102	0.397	0.449
**LC3-II**	0.970 *	0.965 *	0.981 *	0.966 *	0.102	-	0.926 *	0.908 *
**SA-β-gal**	0.827	0.835	0.866	0.895 *	0.397	0.926 *	-	0.962 *
**Ki-67**	0.799	0.790	0.854	0.864	0.449	0.908 *	0.962 *	-

* correlations are significant at *p* < 0.05.

## References

[1] Adamus-Gorka M., Mavroidis P., Lind B.K., Brahme A. (2011). Comparison of dose response models for predicting normal tissue complications from cancer radiotherapy: Application in rat spinal cord. Cancers.

[2] Falk M., Hausmann M. (2020). A Paradigm Revolution or Just Better Resolution-Will Newly Emerging Superresolution Techniques Identify Chromatin Architecture as a Key Factor in Radiation-Induced DNA Damage and Repair Regulation?. Cancers.

[3] Shibata A., Jeggo P.A. (2014). DNA double-strand break repair in a cellular context. Clin. Oncol. (R Coll. Radiol.).

[4] Oh J.M., Myung K. (2022). Crosstalk between different DNA repair pathways for DNA double strand break repairs. Mutat. Res. Genet. Toxicol. Environ. Mutagen..

[5] White R.R., Vijg J. (2016). Do DNA Double-Strand Breaks Drive Aging?. Mol. Cell.

[6] Jiang Y. (2022). Contribution of Microhomology to Genome Instability: Connection between DNA Repair and Replication Stress. Int. J. Mol. Sci..

[7] Sishc B.J., Davis A.J. (2017). The Role of the Core Non-Homologous End Joining Factors in Carcinogenesis and Cancer. Cancers.

[8] Rothkamm K., Barnard S., Moquet J., Ellender M., Rana Z., Burdak-Rothkamm S. (2015). DNA damage foci: Meaning and significance. Environ. Mol. Mutagen..

[9] Bushmanov A., Vorobyeva N., Molodtsova D., Osipov A.N. (2022). Utilization of DNA double-strand breaks for biodosimetry of ionizing radiation exposure. Environ. Adv..

[10] Rahmanian N., Shokrzadeh M., Eskandani M. (2021). Recent advances in gammaH2AX biomarker-based genotoxicity assays: A marker of DNA damage and repair. DNA Repair.

[11] Raavi V., Perumal V., Paul S.F.D. (2021). Potential application of gamma-H2AX as a biodosimetry tool for radiation triage. Mutat. Res. Rev. Mutat. Res..

[12] Osipov A.N., Pustovalova M., Grekhova A., Eremin P., Vorobyova N., Pulin A., Zhavoronkov A., Roumiantsev S., Klokov D.Y., Eremin I. (2015). Low doses of X-rays induce prolonged and ATM-independent persistence of gammaH2AX foci in human gingival mesenchymal stem cells. Oncotarget.

[13] Ulyanenko S., Pustovalova M., Koryakin S., Beketov E., Lychagin A., Ulyanenko L., Kaprin A., Grekhova A., Ozerova A.M., Ozerov I.O. (2019). Formation of gammaH2AX and pATM Foci in Human Mesenchymal Stem Cells Exposed to Low Dose-Rate Gamma-Radiation. Int. J. Mol. Sci..

[14] Markova E., Vasilyev S., Belyaev I. (2015). 53BP1 foci as a marker of tumor cell radiosensitivity. Neoplasma.

[15] Shibata A., Jeggo P.A. (2020). Roles for 53BP1 in the repair of radiation-induced DNA double strand breaks. DNA Repair.

[16] Al Rashid S.T., Dellaire G., Cuddihy A., Jalali F., Vaid M., Coackley C., Folkard M., Xu Y., Chen B.P., Chen D.J. (2005). Evidence for the direct binding of phosphorylated p53 to sites of DNA breaks in vivo. Cancer Res..

[17] Grekhova A.K., Pustovalova M.V., Eremin P.S., Ozerov I.V., Maksimova O.A., Gordeev A.V., Vorobyeva N.Y., Osipov A.N. (2020). Evaluation of the Contribution of Homologous Recombination in DNA Double-Strand Break Repair in Human Fibroblasts after Exposure to Low and Intermediate Doses of X-ray Radiation. Biol. Bull..

[18] Belyaev I.Y. (2010). Radiation-induced DNA repair foci: Spatio-temporal aspects of formation, application for assessment of radiosensitivity and biological dosimetry. Mutat. Res..

[19] Sorokin M., Kholodenko R., Grekhova A., Suntsova M., Pustovalova M., Vorobyeva N., Kholodenko I., Malakhova G., Garazha A., Nedoluzhko A. (2017). Acquired resistance to tyrosine kinase inhibitors may be linked with the decreased sensitivity to X-ray irradiation. Oncotarget.

[20] Banath J.P., Klokov D., MacPhail S.H., Banuelos C.A., Olive P.L. (2010). Residual gammaH2AX foci as an indication of lethal DNA lesions. BMC Cancer.

[21] Vorobyeva N.Y., Babayan N.S., Grigoryan B.A., Sargsyan A.A., Khondkaryan L.G., Apresyan L.S., Chigasova A.K., Yashkina E.I., Guryev D.V., Rodneva S.M. (2022). Increased Yield of Residual γH2AX Foci in p53-Deficient Human Lung Carcinoma Cells Exposed to Subpicosecond Beams of Accelerated Electrons. Bull. Exp. Biol. Med..

[22] Olive P.L. (2011). Retention of gammaH2AX foci as an indication of lethal DNA damage. Radiother. Oncol..

[23] Babayan N., Vorobyeva N., Grigoryan B., Grekhova A., Pustovalova M., Rodneva S., Fedotov Y., Tsakanova G., Aroutiounian R., Osipov A. (2020). Low Repair Capacity of DNA Double-Strand Breaks Induced by Laser-Driven Ultrashort Electron Beams in Cancer Cells. Int. J. Mol. Sci..

[24] Pustovalova M., Astrelina capital Te C., Grekhova A., Vorobyeva N., Tsvetkova A., Blokhina T., Nikitina V., Suchkova Y., Usupzhanova D., Brunchukov V. (2017). Residual gammaH2AX foci induced by low dose x-ray radiation in bone marrow mesenchymal stem cells do not cause accelerated senescence in the progeny of irradiated cells. Aging.

[25] Zorin V., Zorina A., Smetanina N., Kopnin P., Ozerov I.V., Leonov S., Isaev A., Klokov D., Osipov A.N. (2017). Diffuse colonies of human skin fibroblasts in relation to cellular senescence and proliferation. Aging.

[26] Maier A.B., Westendorp R.G., Heemst D.V. (2007). Beta-galactosidase activity as a biomarker of replicative senescence during the course of human fibroblast cultures. Ann. N. Y. Acad. Sci..

[27] Miller I., Min M., Yang C., Tian C., Gookin S., Carter D., Spencer S.L. (2018). Ki-67 is a Graded Rather than a Binary Marker of Proliferation versus Quiescence. Cell Rep..

[28] Sobecki M., Mrouj K., Camasses A., Parisis N., Nicolas E., Lleres D., Gerbe F., Prieto S., Krasinska L., David A. (2016). The cell proliferation antigen Ki-67 organises heterochromatin. Elife.

[29] Sobecki M., Mrouj K., Colinge J., Gerbe F., Jay P., Krasinska L., Dulic V., Fisher D. (2017). Cell-Cycle Regulation Accounts for Variability in Ki-67 Expression Levels. Cancer Res..

[30] Hansen T.E., Johansen T. (2011). Following autophagy step by step. BMC Biol.

[31] Tanida I., Ueno T., Kominami E. (2008). LC3 and Autophagy. Methods Mol. Biol..

[32] Boice A., Bouchier-Hayes L. (2020). Targeting apoptotic caspases in cancer. Biochim Biophys. Acta Mol. Cell Res..

[33] Tsapras P., Nezis I.P. (2017). Caspase involvement in autophagy. Cell Death Differ..

[34] Panneer Selvam S., Roth B.M., Nganga R., Kim J., Cooley M.A., Helke K., Smith C.D., Ogretmen B. (2018). Balance between senescence and apoptosis is regulated by telomere damage-induced association between p16 and caspase-3. J. Biol. Chem..

[35] Eskandari E., Eaves C.J. (2022). Paradoxical roles of caspase-3 in regulating cell survival, proliferation, and tumorigenesis. J. Cell Biol..

[36] di Masi A., Cilli D., Berardinelli F., Talarico A., Pallavicini I., Pennisi R., Leone S., Antoccia A., Noguera N.I., Lo-Coco F. (2016). PML nuclear body disruption impairs DNA double-strand break sensing and repair in APL. Cell Death Dis..

[37] Scully R., Xie A. (2013). Double strand break repair functions of histone H2AX. Mutat. Res..

[38] Lamarche B.J., Orazio N.I., Weitzman M.D. (2010). The MRN complex in double-strand break repair and telomere maintenance. FEBS Lett..

[39] Merighi A., Gionchiglia N., Granato A., Lossi L. (2021). The Phosphorylated Form of the Histone H2AX (gammaH2AX) in the Brain from Embryonic Life to Old Age. Molecules.

[40] Yan W., Shao Z., Li F., Niu L., Shi Y., Teng M., Li X. (2011). Structural basis of gammaH2AX recognition by human PTIP BRCT5-BRCT6 domains in the DNA damage response pathway. FEBS Lett..

[41] Bakkenist C.J., Kastan M.B. (2003). DNA damage activates ATM through intermolecular autophosphorylation and dimer dissociation. Nature.

[42] Firsanov D.V., Solovjeva L.V., Svetlova M.P. (2011). H2AX phosphorylation at the sites of DNA double-strand breaks in cultivated mammalian cells and tissues. Clin. Epigenetics.

[43] Kinner A., Wu W., Staudt C., Iliakis G. (2008). Gamma-H2AX in recognition and signaling of DNA double-strand breaks in the context of chromatin. Nucleic Acids Res..

[44] Ward I.M., Chen J. (2001). Histone H2AX is phosphorylated in an ATR-dependent manner in response to replicational stress. J. Biol. Chem..

[45] Gelot C., Magdalou I., Lopez B.S. (2015). Replication stress in Mammalian cells and its consequences for mitosis. Genes.

[46] Kim K.P., Mirkin E.V. (2017). So similar yet so different: The two ends of a double strand break. Mutat. Res..

[47] Mirman Z., de Lange T. (2020). 53BP1: A DSB escort. Genes Dev..

[48] Derbyshire D.J., Basu B.P., Serpell L.C., Joo W.S., Date T., Iwabuchi K., Doherty A.J. (2002). Crystal structure of human 53BP1 BRCT domains bound to p53 tumour suppressor. EMBO J..

[49] Zimmermann M., de Lange T. (2014). 53BP1: Pro choice in DNA repair. Trends Cell Biol..

[50] Lei T., Du S., Peng Z., Chen L. (2022). Multifaceted regulation and functions of 53BP1 in NHEJmediated DSB repair (Review). Int. J. Mol. Med..

[51] Loughery J., Cox M., Smith L.M., Meek D.W. (2014). Critical role for p53-serine 15 phosphorylation in stimulating transactivation at p53-responsive promoters. Nucleic Acids Res..

[52] Meek D.W. (2009). Tumour suppression by p53: A role for the DNA damage response?. Nat. Rev. Cancer.

[53] Mirzayans R., Andrais B., Scott A., Murray D. (2012). New insights into p53 signaling and cancer cell response to DNA damage: Implications for cancer therapy. J. Biomed. Biotechnol..

[54] Rothkamm K., Kruger I., Thompson L.H., Lobrich M. (2003). Pathways of DNA double-strand break repair during the mammalian cell cycle. Mol. Cell. Biol..

[55] Schmitt C.A. (2007). Cellular senescence and cancer treatment. Biochim. Biophys. Acta.

[56] Ojha R., Ishaq M., Singh S.K. (2015). Caspase-mediated crosstalk between autophagy and apoptosis: Mutual adjustment or matter of dominance. J. Cancer Res. Ther..

[57] Marcotte R., Lacelle C., Wang E. (2004). Senescent fibroblasts resist apoptosis by downregulating caspase-3. Mech. Ageing Dev..

[58] Aliper A.M., Bozdaganyan M.E., Orekhov P.S., Zhavoronkov A., Osipov A.N. (2019). Replicative and radiation-induced aging: A comparison of gene expression profiles. Aging.

[59] Kwon Y., Kim J.W., Jeoung J.A., Kim M.S., Kang C. (2017). Autophagy Is Pro-Senescence When Seen in Close-Up, but Anti-Senescence in Long-Shot. Mol. Cells.

[60] Young A.R., Narita M., Ferreira M., Kirschner K., Sadaie M., Darot J.F., Tavare S., Arakawa S., Shimizu S., Watt F.M. (2009). Autophagy mediates the mitotic senescence transition. Genes Dev..

[61] Campisi J. (2005). Senescent cells, tumor suppression, and organismal aging: Good citizens, bad neighbors. Cell.

[62] Carafa V., Altucci L. (2020). Deregulation of Cell Death in Cancer: Recent Highlights. Cancers.

[63] Li M., You L., Xue J., Lu Y. (2018). Ionizing Radiation-Induced Cellular Senescence in Normal, Non-transformed Cells and the Involved DNA Damage Response: A Mini Review. Front. Pharmacol..

[64] Yang J., Liu M., Hong D., Zeng M., Zhang X. (2021). The Paradoxical Role of Cellular Senescence in Cancer. Front. Cell Dev. Biol..

