# Supplementary material for: Residual Foci of DNA Damage Response Proteins in Relation to Cellular Senescence and Autophagy in X-Ray Irradiated Fibroblasts
Source: Cells. 2023 Apr 21;12(8):1209. doi: 10.3390/cells12081209 (PMC10136818; doi:10.3390/cells12081209)
Supplement: Supplementary file 1 [file cells-12-01209-s001.zip › cells-2303244-supplementary.pdf]

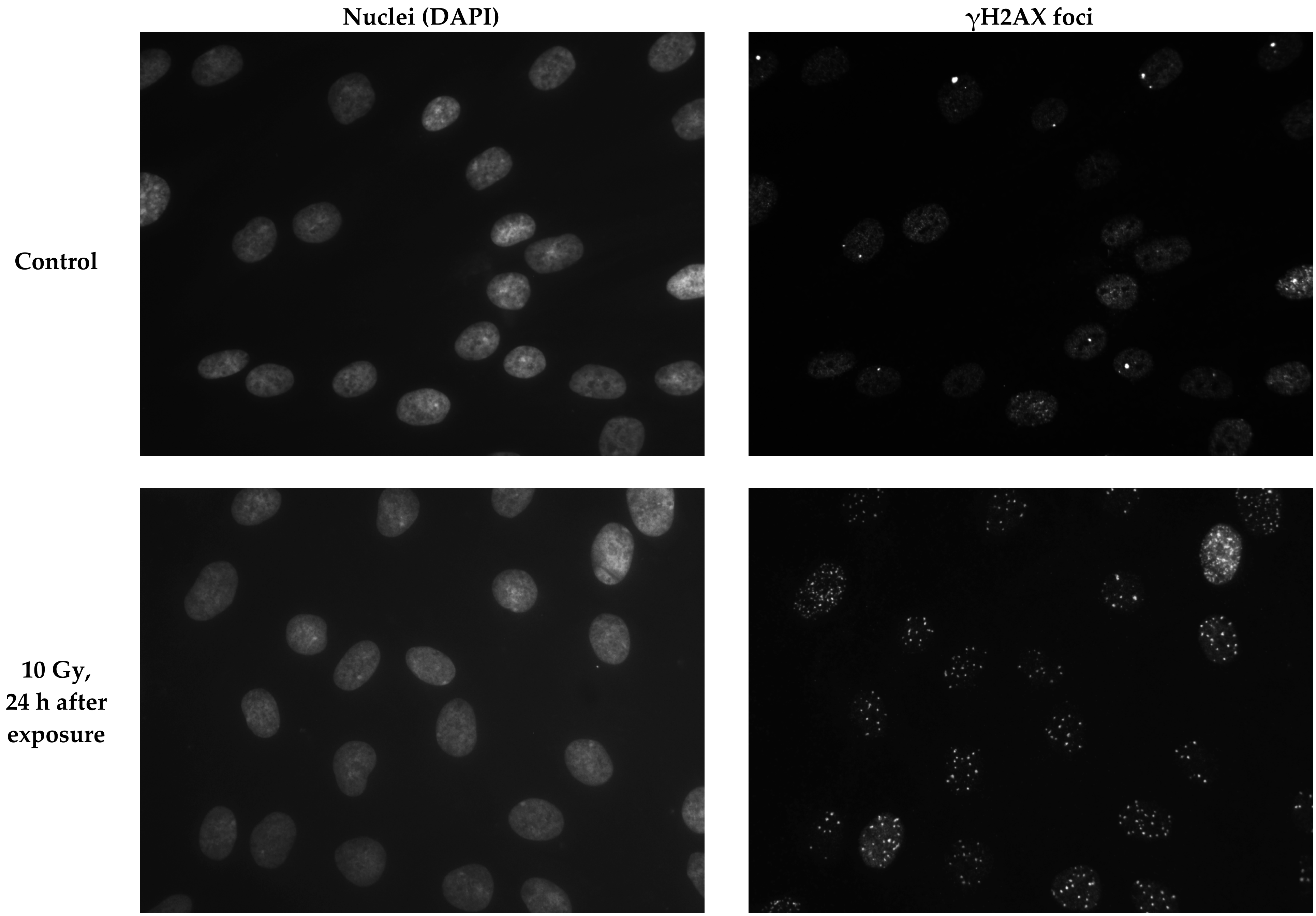
Nuclei (DAPI)
γH2AX foci
Control
10 Gy,
24 h after
exposure

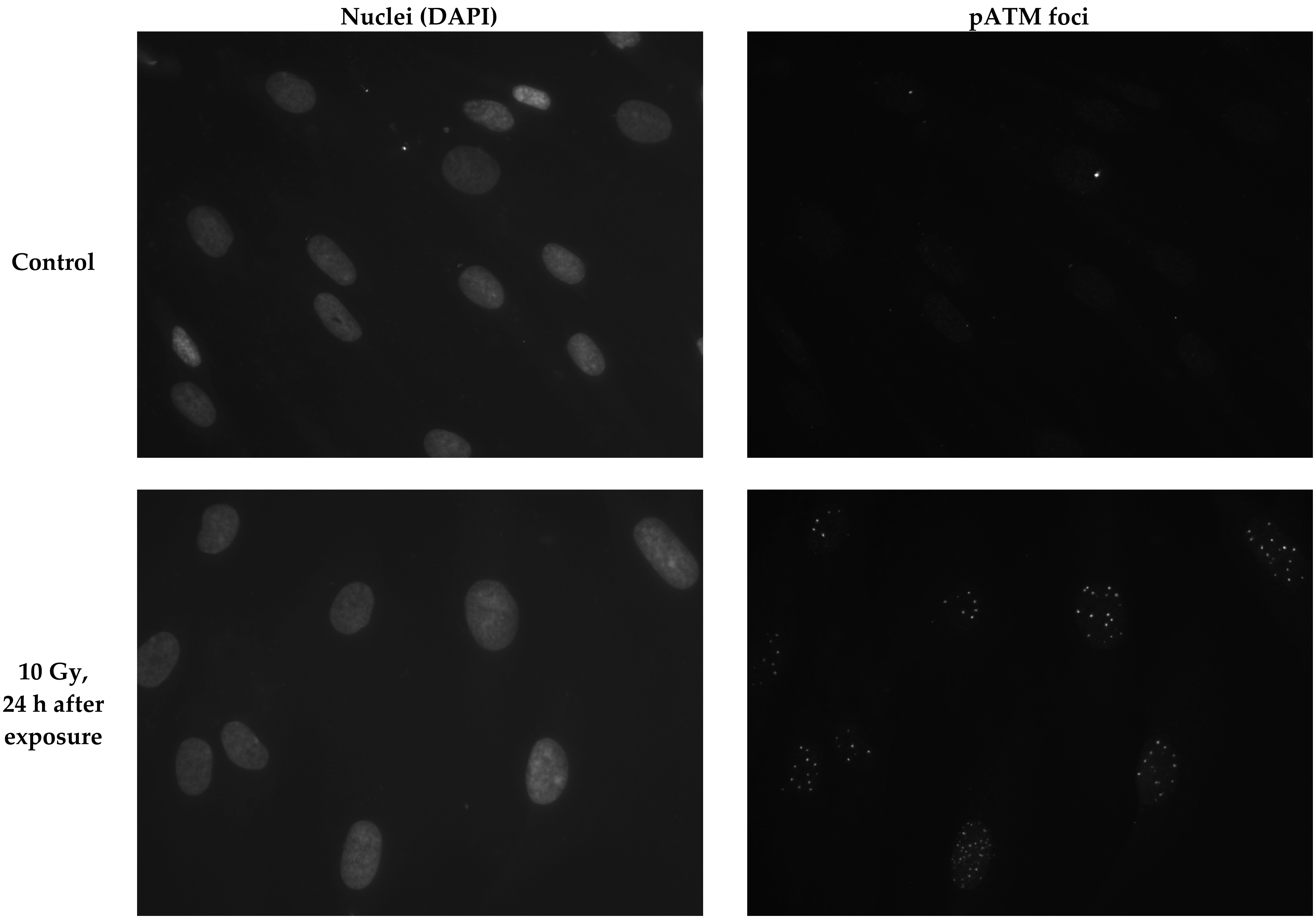
Nuclei (DAPI)
pATM foci
Control
10 Gy,
24 h after
exposure

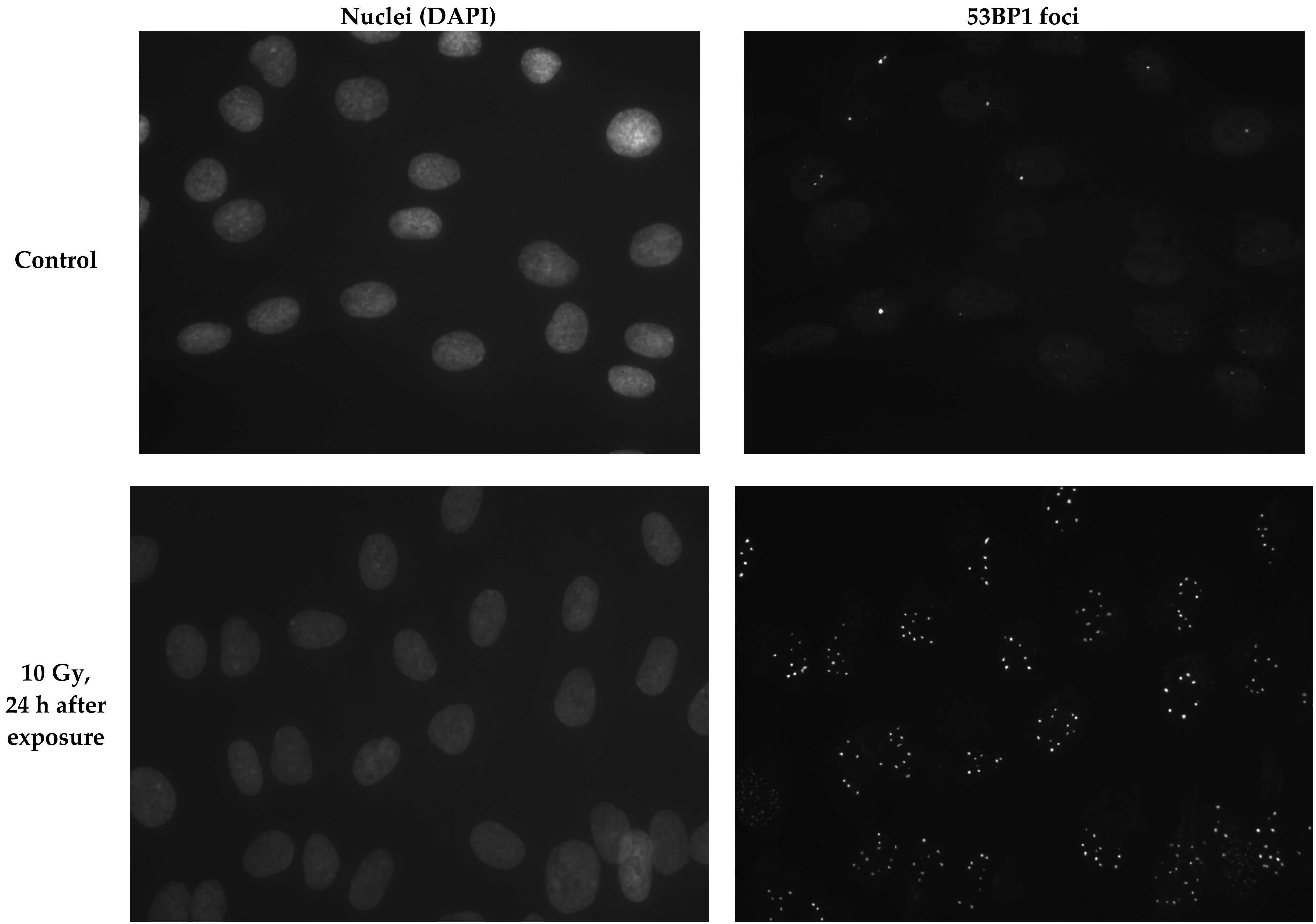
Nuclei (DAPI)
53BP1 foci
Control
10 Gy,
24 h after
exposure

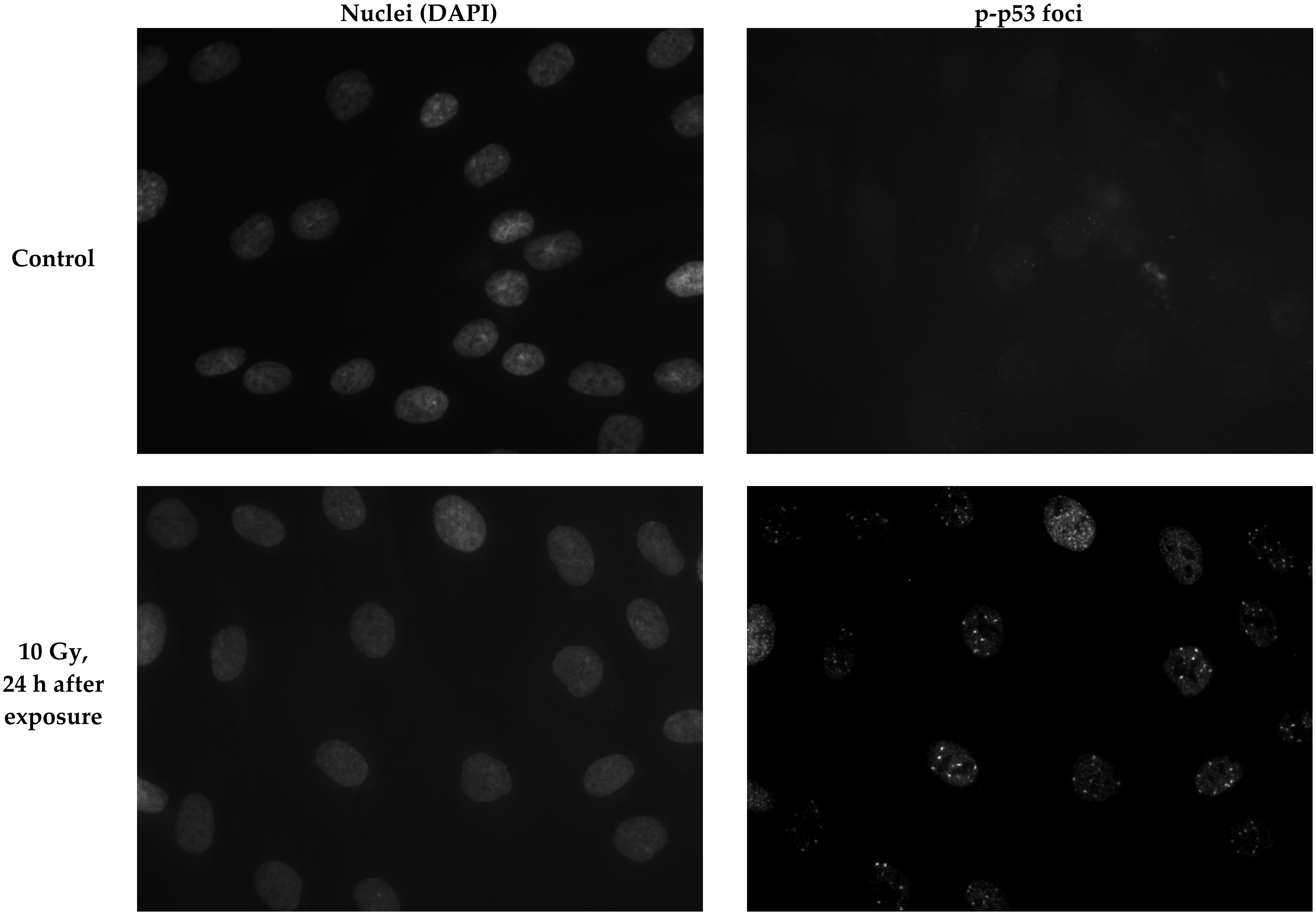
Nuclei (DAPI)
p-p53 foci
Control
10 Gy,
24 h after
exposure

Figure S1. Representative raw microphotographs of immunocytochemically stained control and 10 Gy irradiated fibroblasts.

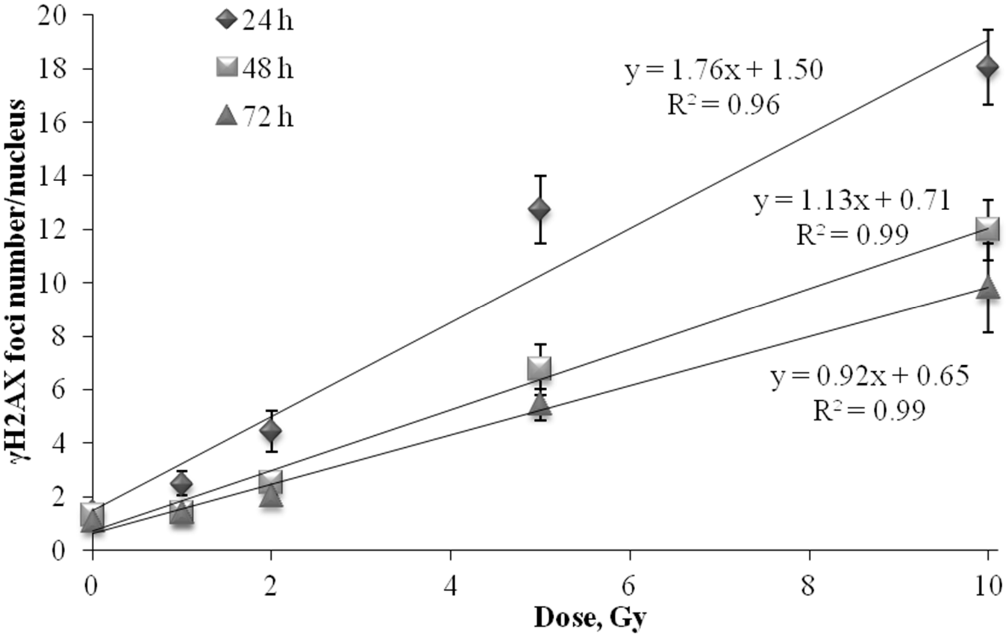
24 h
48 h
72 h
y = 1.76x + 1.50
R² = 0.96
y = 1.13x + 0.71
R² = 0.99
y = 0.92x + 0.65
R² = 0.99
γH2AX foci number/nucleus
Dose, Gy

(a)

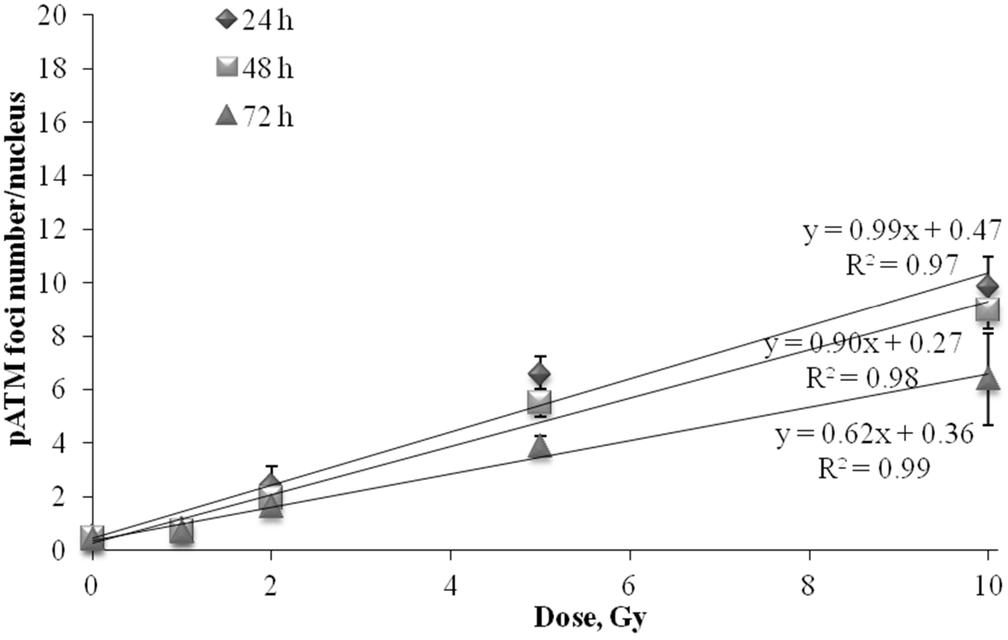
24 h
48 h
72 h
y = 0.99x + 0.47
R² = 0.97
y = 0.90x + 0.27
R² = 0.98
y = 0.62x + 0.36
R² = 0.99
pATM foci number/nucleus
Dose, Gy

(b)

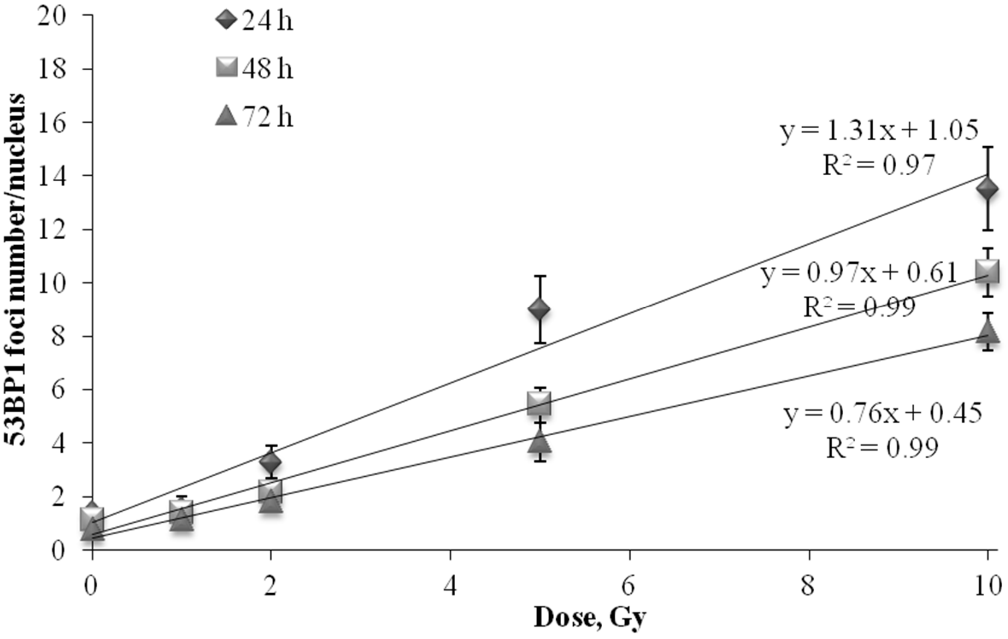

(c)

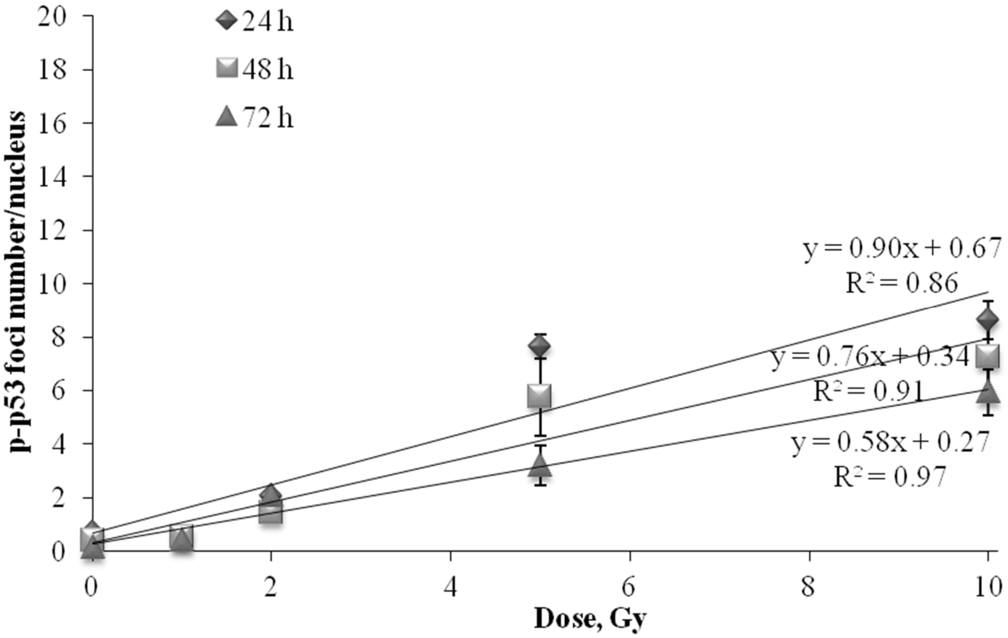

(d)

Figure S2. The dose-response curves and mathematical fits for the residual foci in human fibroblasts 24 h, 48 h and 72 h after irradiation: γH2AX **(a)**, pATM **(b)**, 53BP1 **(c)** and p-p53 (**d)** foci. Data are means ± SE of three independent experiments.
